# Comments on the manuscript entitled: Synchronized aging trajectory of facial and skeletal morphology in Chinese females: implications for rejuvenation strategies

**DOI:** 10.1097/JS9.0000000000004564

**Published:** 2025-12-19

**Authors:** Yuejun Zhao, Leixiang Gong

**Affiliations:** Department of Plastic Surgery, Affiliated Xiaoshan Hospital, Hangzhou Normal University, Hangzhou, China

*Dear Editor,*

A research paper entitled “Comments on the manuscript entitled: Synchronized aging trajectory of facial and skeletal morphology in Chinese females: Implications for rejuvenation strategies” recently published in the International Journal of Surgery has caught our attention[1]. This study differentiates itself from conventional static measurements by employing advanced geometric morphometrics (GM). The identification of the dynamic three-dimensional processes of facial skeletal aging enables the construction of facial aging trajectories, thereby providing data references for personalized rejuvenation treatments targeting facial aging. Zhong *et al* found that facial aging and skeletal aging are significantly correlated, yet not synchronized temporally. The results of the research indicate that the onset of facial aging occurs at the age of 30, with a subsequent acceleration from the age of 35. This primarily manifests as a shortening of the midface height, retraction of the infraorbital rim and platysma muscle, asymmetrical midface ptosis, redistribution of fat, and elongation of the philtrum. These findings are both timely and significant. While the rigorous efforts and valuable contributions of this study are deeply appreciated, some constructive suggestions are offered for further refinement. This commentary adheres to the TITAN Guidelines 2025 for the declaration and use of artificial intelligence in scientific publications, and no AI tools were used in the design, analysis, or writing of this work[2].

First, for the population included in the study. This study included 348 healthy Chinese women, categorized into six age groups: 18–20 years (28, 8.05%), 20–25 years (71, 20.40%), 25–30 years (131, 37.64%), 30–35 years (71, 20.40%), 35–40 years (39, 11.20%), and over 40 years (8, 2.30%).

This study utilized a cross-sectional dataset, with the inferred trajectories representing population-level variations. Consequently, the accelerated aging points proposed here may be subject to cohort effects and sampling differences[3]. This study found that facial aging accelerates from the age of 35 to 40. However, the proportion of participants aged 35 and above – particularly those over 40 – was relatively low. This may lead to underestimation or excessive smoothing of skeletal morphological changes in individuals aged 35 and above, thereby compromising the accuracy of modelling aging trajectories. Therefore, incorporating more participants and conducting longitudinal studies would be more conducive to strengthening causal inferences between bone and facial aging. Furthermore, the participants in this study were recruited from a single center and were exclusively Chinese women, which introduces potential selection bias and population bias[4].

Second, there are potential confounding factors. This study employed precise inclusion and exclusion criteria to eliminate the influence of congenital dysplasia, maxillofacial trauma, and surgical factors on maxillofacial skeletal morphology. However, certain key factors influencing facial and skeletal aging are overlooked, such as BMI, lifestyle habits (sun exposure, smoking, alcohol consumption, and daily nutritional intake), and dental health and occlusal function^[5-7]^.

Finally, the focal point of this study was the superficial 3D facial surface and the bony CT structures of the face. Nevertheless, the process of facial aging is characterized by its multifaceted nature. The absence of measurements for key anatomical layers, such as the deep fat pad, superficial musculoaponeurotic system, and crucial facial ligaments and muscles, imposes certain limitations on research into the mechanisms of aging^[8-10]^.

In summary, the study by Zhong *et al* employed advanced GM techniques to visualize age-related morphological changes and generate aging trajectories, thereby demonstrating a significant association between facial aging and skeletal alterations. This research constitutes an innovative and significant step toward establishing more precise aging trajectories, thereby laying a solid foundation for the early prevention of facial aging. Our suggestions are merely intended to further refine an already excellent piece of research. We eagerly anticipate more innovative work from the authors in the future.

## Data Availability

No other datasets were generated during and/or analyzed during the current study. All the information is available with the manuscript.
